# Spontaneous Hemothorax in Neurofibromatosis Type 1: A Surgical Case Report

**DOI:** 10.7759/cureus.89615

**Published:** 2025-08-08

**Authors:** Naoya Ishibashi, Ryuga Yabe, Kazunori Ueda, Ryo Nonomura, Yutaka Oshima, Takanobu Sasaki, Takafumi Sugawara, Hiromichi Niikawa

**Affiliations:** 1 Thoracic Surgery, Tohoku Medical and Pharmaceutical University, Sendai, JPN; 2 Thoracic Surgery, Tohoku Medical And Pharmaceutical University, Sendai, JPN

**Keywords:** acute chest pain, chest pain, neurofibromatosis type 1 (nf-1), thoracic surgery, von recklinghausen’s disease

## Abstract

Neurofibromatosis type 1 (NF-1) is an autosomal dominant disorder associated with vascular abnormalities, including spontaneous hemothorax and arterial aneurysms. We present a rare case of spontaneous hemothorax in which an apparently hemostatic sub-pleural hematoma began to bleed again after the patient was repositioned. A 47-year-old man with NF-1 presented with the sudden onset of left-sided chest pain. Contrast-enhanced chest computed tomography revealed a massive left-sided hemothorax with a tortuous intercostal artery and an adjacent hematoma. The patient underwent emergency video-assisted thoracoscopic surgery (VATS), which revealed extensive intrathoracic clots and a subpleural hematoma near the aortic arch. Although no active bleeding was observed intraoperatively, hemostatic agents were applied to the suspected site. However, massive rebleeding occurred following patient repositioning, prompting an urgent re-thoracotomy. Active bleeding from the intercostal artery was identified and successfully controlled. The patient recovered without recurrence of hemothorax. Spontaneous hemothorax in patients with NF-1 warrants early recognition and surgical intervention. Vascular reconstruction is often challenging due to arterial fragility, underscoring the importance of careful intraoperative hemostasis and treatment selection. Furthermore, surgeons should be aware that temporary hemostasis may be achieved due to tamponade by a subpleural hematoma, potentially masking an underlying vascular injury. Careful intraoperative assessment and readiness for reintervention are essential in managing such cases.

## Introduction

Neurofibromatosis type 1 (NF-1) is an autosomal-dominant disorder characterized by café-au-lait spots, cutaneous and plexiform neurofibromas, and a spectrum of vascular abnormalities [1]. Vascular lesions, including aneurysms, stenoses, arteriovenous malformations, and spontaneous ruptures, are well-recognized manifestations of NF-1 that can involve virtually any medium- or large-sized artery. For example, the intercostal and subclavian branches that supply the thoracic cavity may be affected. The pathophysiology of these vascular complications includes arterial wall compression by adjacent tumors, intimal invasion by fibroma cells, and wall thinning secondary to abnormal smooth-muscle proliferation [2]. Hemorrhagic events are therefore not uncommon; in a surgical series of neurofibroma resections, perioperative bleeding occurred in 46.8% of patients with NF-1 [3]. Although spontaneous hemothorax is far less frequent, it is potentially fatal and demands prompt recognition and management. We describe a patient with NF-1 who developed spontaneous hemothorax; apparent intraoperative hemostasis was followed by postoperative re-bleeding, underscoring the risk of concealed bleeding temporarily masked by tamponade.

## Case presentation

**Figure 1:**
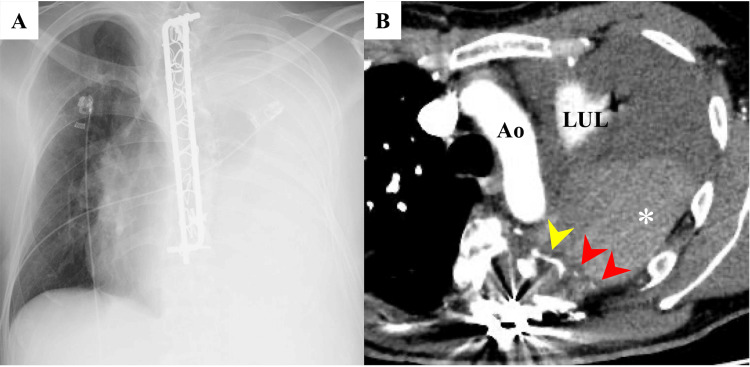
Chest X-ray (A) and chest computed tomography (B) A: Chest X-ray showing an opaque left hemithorax caused by massive pleural effusion (hemothorax) on admission. Linear metallic opacities along the thoracic spine correspond to prior scoliosis instrumentation. B: Contrast-enhanced chest computed tomography revealing a clot in the left thoracic cavity (asterisk), a hematoma along the left margin of the vertebral body (red arrowhead), and a tortuous intercostal artery (yellow arrowhead). LUL: Left upper lobe, Ao: Aortic arch

A 47-year-old man with a known history of neurofibromatosis type 1 (NF-1) presented to a local emergency department with a sudden onset of left-sided pain. At that time, his vital signs were notable for a heart rate of 140-150 beats per minute, a systolic blood pressure of 100-120 mmHg, and an oxygen saturation of 100% while receiving oxygen via face mask at 3 liters per minute. The left thoracic drain was inserted, and it yielded bloody pleural fluid. The patient was referred to the receiving hospital. On arrival, his blood pressure was stable, and laboratory tests revealed hemoglobin of 8.4 g/dL, hematocrit of 24.4%, and red blood cell count of 2.61 × 10^6/μL. The coagulation parameters were normal. A chest X-ray demonstrated an opaque left hemithorax (Figure 1A), whereas contrast-enhanced computed tomography revealed a tortuous intercostal artery branching from the posterior aspect of the aortic arch with suspected surrounding hematoma formation (Figure 1B). Based on these findings, emergency surgery was indicated.

**Figure 2:**
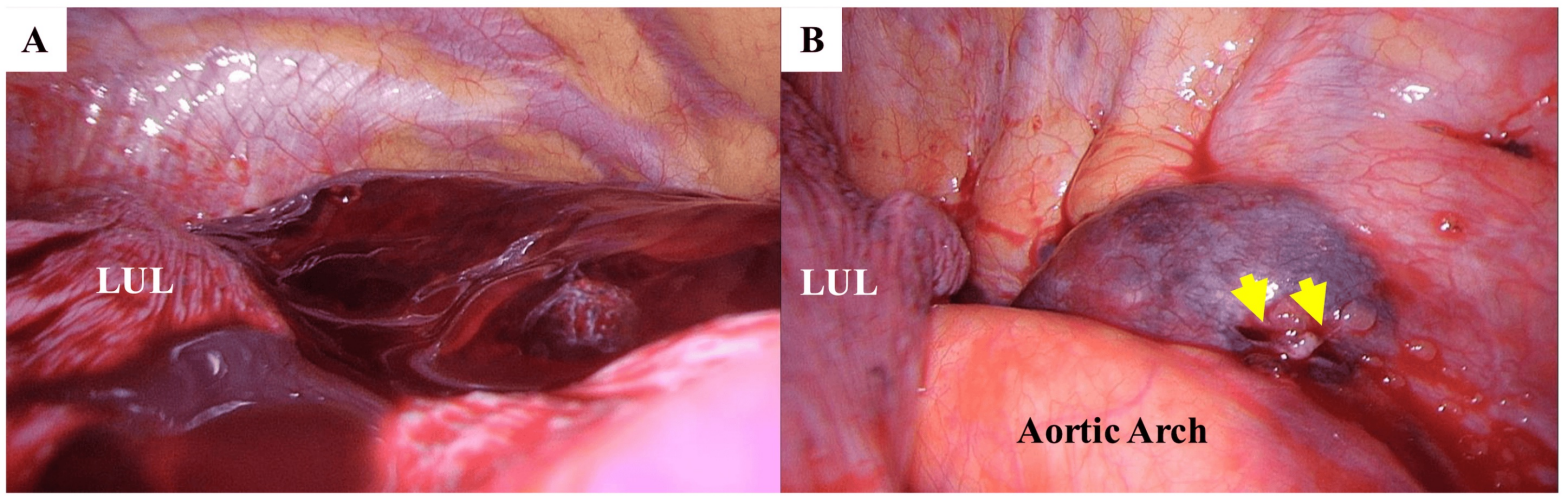
Intraoperative findings (A, B) A: Intraoperative findings showing extensive blood and clots in the left thoracic cavity. B: After removal of the blood and clots, a subpleural hematoma is observed on the dorsal aspect of the aortic arch. A laceration (yellow arrow) is visible on its surface, although no active bleeding was noted at that time. LUL: Left upper lobe

The operation was performed with the patient in the right lateral decubitus position using complete video-assisted thoracoscopic surgery (VATS) with two ports and one window (30 mm). The thoracic cavity was filled with blood and clots. After their removal, a subpleural hematoma was identified posterior to the aortic arch. The hematoma surface had a small laceration but no active bleeding; therefore, hemostatic agents were applied, a chest drain was inserted, and the procedure was completed (Figures 2A, 2B).

Immediately after the patient was repositioned from the lateral to supine position, 300 mL of bloody pleural fluid rapidly drained from the chest tube. The patient immediately returned to the right lateral position, and the thoracic cavity was re-examined. Significant bloody pleural fluid and bleeding from the hematoma were observed. Compression of the hematoma failed to achieve hemostasis. After hematoma removal, pulsatile bleeding from the exposed intercostal artery was identified. The incision was extended, and the intercostal artery was sutured using four 4-0 Prolene stitches. Intraoperative blood transfusion included 12 units of packed red blood cells and 8 units of fresh frozen plasma, with an estimated blood loss of 6,600 mL.

The patient was admitted to the postoperative intensive care unit and underwent blood sampling, which revealed a platelet count of 68,000/μL, a decrease from the preoperative value (211,000/μL). Therefore, 15 units of platelet transfusion were administered to correct the bleeding tendency associated with thrombocytopenia. His hemodynamic status stabilized, and he was transferred out of the ICU the following day. The chest drain was removed on postoperative day 7, and the patient was discharged on postoperative day 11. No recurrence of hemothorax was observed thereafter.

## Discussion

Pathophysiology of vascular lesions in NF-1

The pathological characteristics of arterial walls in NF-1 include ischemia due to the compression of the adventitia by fibroma cells, direct invasion of fibroma cells into the intima, and thinning of the intima due to smooth muscle proliferation [1]. These changes significantly increase the risk of serious vascular lesions, such as aneurysms and stenosis, in patients with NF-1 [2]. Vascular abnormalities have been documented in almost every arterial territory -- renal, intracranial, carotid-vertebral, subclavian, mesenteric, and, more rarely, intercostal arteries reported to date [4]. Ischemia due to stenosis or compression of the surrounding organs by aneurysms may cause pain or dyspnea, which can be the initial presentation leading to diagnosis [5-7]. Hemorrhage due to dissection or vascular wall rupture has also been reported, with an incidence of approximately 3.6% [8].

Comparison with previous reports

Most hemorrhagic events occur when the arterial wall cannot withstand blood pressure and ruptures; however, external factors may contribute to the rupture, as exemplified by a case in which mechanical stimulation from a kyphotic cervical spine led to the rupture of a fragile vertebral artery aneurysm [5]. Hemothorax typically presents with chest pain and dyspnea that may be refractory to analgesics, with chest X-ray and CT demonstrating pleural effusion [9]. Similarly, our patient presented with significant pleural effusion and chest pain that were unresponsive to analgesics. Contrast-enhanced CT revealed a tortuous vessel and hematoma but did not demonstrate a clear aneurysm or extravasation of the contrast media.

Comparison of treatment strategies

Although catheter-based interventions have been reported as effective for aneurysms in NF-1 [5,10-13], there are also reports of aneurysm rupture induced by treatment stimulation and cases in which vascular reconstruction attempts failed due to fragile vessel walls, necessitating ligation [2]. Therefore, the indications for catheter-based treatment in hemodynamically stable patients with unruptured aneurysms should be carefully evaluated. In our case, no clear aneurysm was identified, and emergency surgery was performed for hematoma removal and hemostasis. Although catheter-based interventions achieve hemostasis, the intrathoracic hematomas cannot be adequately removed by drainage alone and may impede lung expansion. Consequently, elective hematoma removal should be considered after hemodynamic stabilization to preserve respiratory function [9].

Clinical lessons from the present case

Apparent intraoperative hemostasis in this case was likely due to a combination of transient hypotension under general anesthesia, compression by a pre-existing subpleural hematoma, and decreased intrathoracic pressure in the lateral decubitus position, all of which may have temporarily masked ongoing arterial bleeding. This pseudohemostasis led to the erroneous impression that bleeding had ceased. Such intraoperative findings highlight the limitations of relying solely on visual assessment to confirm hemostasis. The subsequent rebleeding after postoperative repositioning represents an exceptionally rare clinical course not commonly described in previous reports. Although Koike et al. reported a case of tension hemothorax in NF-1, no reports have described rebleeding triggered by the release of intraoperative temporary hemostasis [12].

When rebleeding occurs following an initial VATS procedure, prompt conversion to thoracotomy and direct surgical hemostasis should be considered. In our case, the bleeding was successfully controlled via direct suture ligation under open thoracotomy. Surgeons must maintain a high index of suspicion for hidden bleeding sources beneath hematomas and should not hesitate to perform secure suture ligation during the initial operation, especially in patients with NF-1 and fragile vasculature.

## Conclusions

Spontaneous hemothorax in NF1 can originate from fragile, abnormal intercostal arteries concealed beneath subpleural hematomas. When such an artery is identified, or even suspected, definitive control at the initial operation is essential, because apparent hemostasis may be lost after lung re-expansion or patient repositioning, resulting in life-threatening re-bleeding. Therefore, surgical exploration and targeted ligation of the abnormal intercostal artery are critical to prevent recurrent hemorrhage.
